# Italian experience with rVIII-single chain: a survey of patients with haemophilia A and their physicians

**DOI:** 10.1007/s11239-021-02599-w

**Published:** 2021-11-13

**Authors:** Alessandra Borchiellini, Giancarlo Castaman, Giulio Feola, Antonietta Ferretti, Paola Giordano, Matteo Luciani, Giuseppe Malcangi, Maurizio Margaglione, Angelo Claudio Molinari, Berardino Pollio, Angiola Rocino, Cristina Santoro, Michele Schiavulli, Ezio Zanon

**Affiliations:** 1 Centro di Riferimento Regionale Malattie Emorragiche e Trombotiche dell’adulto Ematologia U Città della Salute, Torino, Italy; 2 Department of Oncology, Center for Bleeding Disorders and Coagulation, Careggi University Hospital, Florence, Italy; 3 Centro Emofilia di Vallo della Lucania, Salerno, Italy; 4 Department of Translational and Precision Medicine, Sapienza University, Rome, Italy; 5 Paediatric Section, Department of Biomedicine and Human Oncology, University of Bari, Bari, Italy; 6 Oncohematology Department Bambino, Gesù Pediatric Hospital, Rome, Italy; 7 UOSD Emofilia e Trombosi Azienda Ospedaliero Universitaria Policlinico di Bari, Bari, Italy; 8 Genetica Medica Dip.to Medicina Clinica e Sperimentale Università di Foggia, Foggia, Italy; 9 Regional Reference Center for Hemorrhagic Diseases, Giannina Gaslini Children’s Hospital, Genoa, Italy; 10 Centro di Riferimento Regionale Malattie Emorragiche e Trombotiche Ereditarie in età pediatrica, S.S.D. Medicina Trasfusionale Materno-Infantile-Traumatologica, Azienda Ospedaliera Citta’ Della Salute e della Scienza-Ospedale Infantile Regina Margherita, Turin, Italy; 11 Hematology Unit-Haemophilia and Thrombosis Centre, Ospedale del Mare, Napoli, Italy; 12 Hematology, University Hospital Policlinico Umberto I, Rome, Italy; 13 Dipartimento di Oncologia, Centro di Riferimento Regionale per le Emocoagulopatie, AORN Santobono Pausilipon, Napoli, Italy; 14 Haemophilia Centre, Department of Medicine, University Hospital of Padua, Padua, Italy

**Keywords:** Haemophilia A, rVIII-SingleChain, Injection frequency

## Abstract

rVIII-SingleChain is indicated for treatment and prophylaxis of bleeding in patients with haemophilia A (HA). The safety and efficacy of rVIII-SingleChain have previously been shown in the AFFINITY clinical trial programme. This survey evaluated clinical experience following a switch to rVIII-SingleChain from the perspective of both physicians and patients. A web-based survey (July–September 2019) involving 14 Haemophilia Treatment Centres (HTCs) collected data about HA patients who were under treatment with rVIII-SingleChain for ≥ 12 months, as reported by their physicians. In addition, about half of these patients were separately interviewed. Out of 91 patients receiving rVIII-SingleChain in the 14 participating HTCs, 48 had been treated for ≥ 12 months; among those 48, 38% were ≤ 18 years, 37% 19–40 years and 25 % ≥ 41 years; 73% of them had severe HA and 85% were being treated with prophylactic therapy. Twenty-six patients accepted to be separately interviewed: mean age was 30 years; 62% had severe HA and 85% were receiving prophylaxis. Focusing on those patients who were already in prophylaxis with prior FVIII (all but one with recombinant factors), infusion frequency was significantly reduced from 3–2 per week following the switch to rVIII-SingleChain (mean, 2.74 vs. 2.44, respectively; p=0.013), as reported by physicians; the rate of patients needing 3 infusions per week dropped from 74% with previous products to 44% with rFVIII-SingleChain. The annual mean factor consumption was 4740 IU/Kg (median, 4500 IU/Kg; min, 2.215 IU/Kg; max, 7.200 IU/Kg) with prior product and 4320 IU/Kg (median, 4320 IU/Kg; min, 2.215 IU/Kg; max, 6.646 IU/Kg) with rVIII-SingleChain. Both physicians and patients reported a significant reduction in annual total bleeding rates with rVIII-SingleChain compared with prior product (mean 2.15–0.96 and 2.46–0.71 events/year, p = 0.031 and p = 0.018, respectively). Mean satisfaction ratings (from 1; dissatisfied, to 5; very satisfied) for rVIII-SingleChain were quite high for both physicians (4.14, 86% satisfied/very satisfied) and patients (4.18, 86% satisfied/very satisfied). This survey suggested that switching to rVIII-SingleChain allowed patients to reduce their injection frequency without increasing factor consumption or compromising clinical results. Both physicians and patients reported a positive experience with rVIII-SingleChain after 1 year of treatment.

## Highlights

- Clinicians indicated rVIII-SingleChain as a reliable choice with a favorable pharmacokinetic profile.
- Switching to rVIII-SingleChain allowed patients to reduce their injection frequency without increasing factor consumption or compromising clinical results.
- Both physicians and patients reported a positive experience with rVIII-SingleChain after 1 year of treatment.
- Results of this real-world study validate clinical trials’ results in the daily clinical practice.

## Introduction

Haemophilia A (HA) is an X-linked congenital bleeding disorder resulting from mutations in the gene encoding the coagulation factor VIII (FVIII) [1]. Regular prophylactic factor VIII replacement is currently the standard treatment for patients with severe haemophilia A (defined as FVIII levels < 1 IU/dL), and its introduction has greatly improved the prognosis, life expectancy, and quality of life (QoL) of subjects with haemophilia, particularly preventing spontaneous bleeding episodes into the joints or the muscles, and the developing of haemophilic arthropathy [2, 3]. However, issues related to the burden of treatment, due to the need for frequent injections, and to the serious complication of inhibitor development, still calls for the need of effective new agents with improved stability and reduced immunogenicity.

rVIII-SingleChain is a novel recombinant FVIII (rFVIII) molecule designed to provide improved stability compared with other available rFVIII, through the formation of a covalent linkage between the light and heavy chains of FVIII [4]. The resulting single-chain rFVIII molecule demonstrated enhanced PK parameters [5], and higher affinity for von Willebrand factor (VWF). VWF helps stabilizing FVIII and protects it from premature clearance, potentially also playing a role in reducing the development of inhibitors by limiting the recognition of rFVIII by antigen-presenting cells [4]. Thanks to its innovative features, rVIII-SingleChain allows for less frequent prophylaxis regimes (i.e. 2–3 times per week) compared to standard half-life (SHL) rFVIII products, which typically require 3–4 infusions per week, with major advantages in terms of burden of care and compliance to treatment for patients [6]. Since even most effective current prophylactic regimens may not completely prevent joint disease, in a long-term perspective adherence to therapy has important implications for the functional outcome of haemophilic patients.

The efficacy and safety of rVIII-SingleChain in previously treated adult/adolescent and children patients with severe HA were evaluated in the AFFINITY series of clinical trials, involving a total of 259 patients including 84 children [7, 8]. RVIII-SingleChain when administered on-demand or in a prophylaxis regimen demonstrated to effectively control bleeding episodes, with high treatment success rates and good/excellent haemostatic efficacy in more than 90% of cases, and to be associated with low annualized bleeding rates in patients on prophylaxis. Surgical haemostasis with rVIII-SingleChain was also effective during major surgeries in adult and adolescent patients and rated as excellent in 94% of surgeries [7]. None of the participant to the trials developed FVIII inhibitors [7, 8].

A key point in the clinical use of rVIII-SingleChain is the dosing regimen in patients receiving routine prophylaxis. Confirming the benefits of rVIII-SingleChain improved stability, a consistent proportion of adult or children patients enrolled in the two pivotal trials were able to reduce their injection frequency compared with their pre-study regimen [7, 8]. These findings were recently confirmed by real-world studies performed in Europe (Germany) and United States, which reported reduced dosing frequency and consumption compared with prior treatment in patients with haemophilia A who switched to rVIII-SingleChain prophylaxis, with similar bleeding rates [6, 9].

In the present study, we report a national survey among Italian physicians and patients treated for at least 12 months with rVIII-SingleChain, with the aim of obtaining a real-world picture of doctors’ and patients’ experiences and perceptions about the drug and collecting data on the number of injections, consumption of FVIII, and number of bleeding episodes in patients receiving rVIII-SingleChain. The results presented in this paper offer a quite comprehensive picture of rVIII-SingleChain good clinical results in real life.

## Methods

A web-based survey was designed to collect data about HA patients who were under treatment with rVIII-SingleChain for ≥ 12 months, as reported by their physicians. In addition, part of these patients were separately interviewed. The survey, involving 14 Italian Haemophilia Treatment Centres (HTCs), was carried out between July and September 2019, with the support of IQVIA^TM^, who also performed all data analyses.

### Survey design

**Fig. 1:**
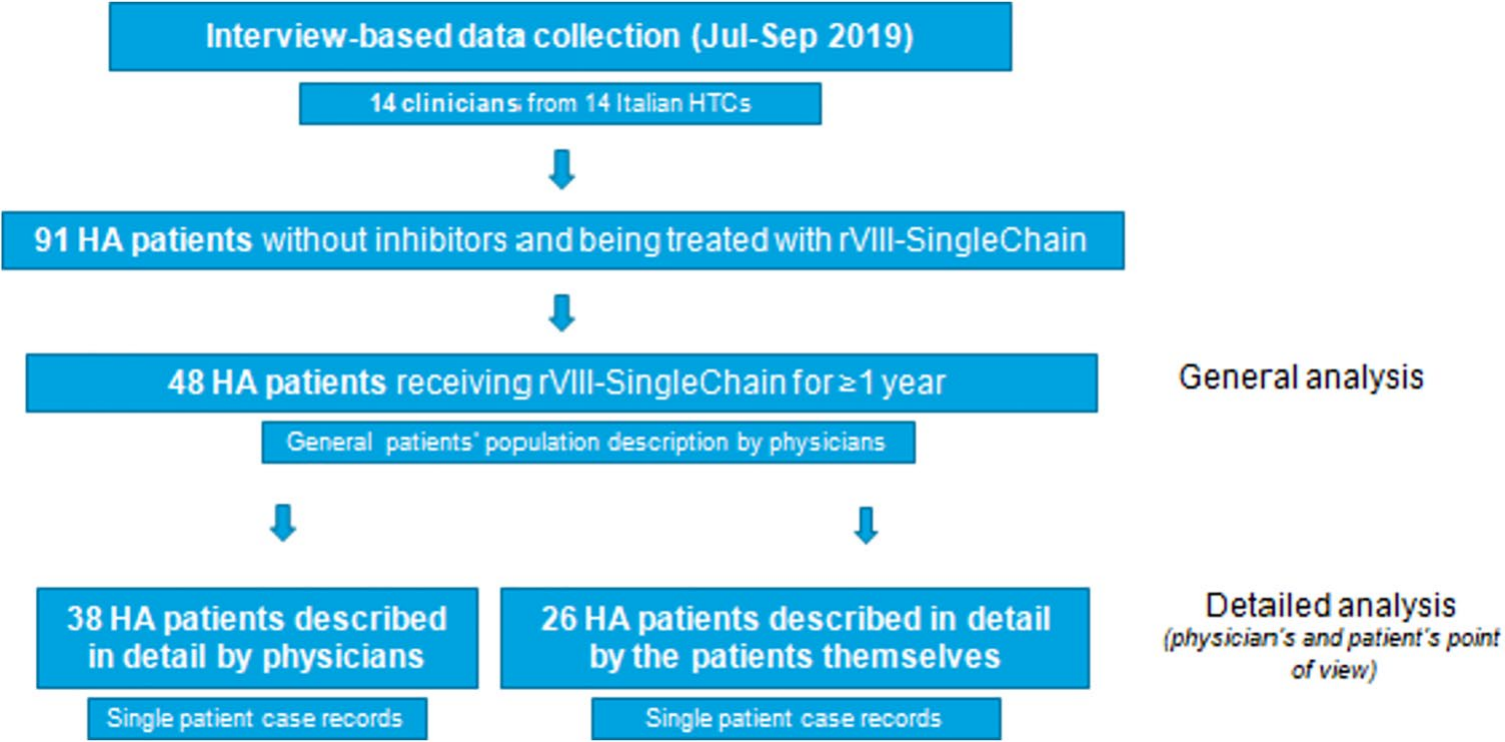
Survey design and patients’ disposition

The data collection included a *physicians’ phase* and a *patients’ phase*. For the *physicians’ phase*, 14 specialists from 14 HCTs were questioned by computer-assisted web interviewing (CAWI) interviews lasting 30 min. Patients treated with rVIII-SingleChain for at least 12 months could be included in the analysis. The questionnaire comprised two sections: a general section, reporting a comprehensive description of physician’s experience with HA patients receiving rVIII-SingleChain (cohort A:48 patients), and a section comprising detailed records of single patients treated with rVIII-SingleChain, when available (cohort B: 38 patients, one case record for each patient). For the *patients’ phase*, physicians sent a web link to their patients receiving rVIII-SingleChain for ≥ 12 months, 26 of whom anonymously completed an on-line questionnaire about their experience with rVIII-SingleChain (cohort C:26 patients). The on-line questionnaires for doctors and patients were designed to allow for a comparison between the clinician’s point of view and the patient’s direct experience, mostly including analogous questions. However, the population of patients interviewed did not necessarily correspond to that described by the physicians (Fig. 1).

### Data collected

The main outcomes assessed by the survey were: description of patients treated with rVIII-SingleChain for at least 12 months (general description of cohort A patients and detailed description of cohort B patients by clinicians from clinical records; detailed description of cohort C using records compiled by the patients themselves), including treatment prior to prophylaxis with rVIII-SingleChain and pharmacokinetic assessment; changes observed after 12 months of therapy with rVIII-SingleChain in terms of dosage regimen, FVIII consumption, and rate of annual total bleeds (ABR) and joint bleeds (AJBR); level and areas of satisfaction with rVIII-SingleChain by the physicians’ and patients’ perspective (satisfaction ratings, from 1, dissatisfied; to 5, very satisfied); patient’s general experience with the disease and the prophylactic therapy, relationship with the Haemophilia Centre, level of information on haemophilia.

### Statistical analysis

Standard descriptive statistics were used to analyse the survey results. Categorical data were described by numbers and percentages, and continuous variables by mean or median and ranges. To assess differences in outcome before and after the switch to rVIII-SingleChain, the Student t test, specifically indicated for small sample sizes (< 30 cases), was employed. A P value < 0.05 was considered statistically significant. All statistical analyses were performed by IQVIA^TM^.

## Results

### General description of patients’ population

A total of 14 physicians from 14 Italian HTCs were enrolled in the study. Overall, they reported a total of 91 HA patients without inhibitors being treated with rVIII-SingleChain under the Centre’s care. Out of these, 48 patients (53%) had been receiving the drug for at least 12 months (mean follow-up: 15.8 months), and were included in the general population (cohort A). Demographic and clinical characteristics of all enrolled patients are summarized in Table 1.

**Table 1:** Demographic and clinical characteristics of the total population of patients (cohort A)

Characteristics	Number (%)
Number of patients	48
Age	
0–12 years	10 (21)
13–18 years	8 (17)
19–40 years	18 (37)
41–65 years	9 (19)
> 65 years	3 (6)
Haemophilia severity	
Mild	3 (6)
Moderate	10 (21)
Severe	35 (73)
Treatment regimen	
On-demand	7 (15)
Prophylaxis	41 (85)
Previous treatment (prophylaxis pts. n=41)	
PUPs	1 (2)
PTPs on-demand	5 (12)
PTPs on regular prophylaxis	35 (86)

*PUPs* previously untreated patients, *PTPs* previously treated patients

Out of these 48 patients, 38% were ≤ 18 years, 37 % 19–40 years and 25 ≥41 years. Forty-one/48 patients (85 %) were on a regular prophylaxis regimen with rVIII-SingleChain; of these, 33 (80%) had a severe form of haemophilia and 8 (20%) a moderate form. The majority of patients on prophylaxis (n=35, 86%) were already treated with a prophylactic regimen before the switch to rVIII-SingleChain; 5 (12%) received an on-demand therapy and 1 patient (2%) was previously untreated (PUP) (Table 1). The most frequent previous medication used for prophylaxis by the 35 patients was a full length 2nd generation rFVIII from baby hamster kidney (BHK) cells (Helixate NG) (66 ); 17% were treated with 3rd generation rFVIII; 14% with other 1st/2nd generation rFVIII, and one patients (3%) with a plasma–derived FVIII product (3 %). No one used extended half life (EHL) products

### PK monitoring

Among the total population of 48 patients treated with rVIII-SingleChain for at least 1 year (cohort A), 56% of cases (27 patients) underwent pharmacokinetic (PK) studies in the last 12 months during their transition to the new drug or later to optimize their rVIII-SingleChain treatment. 21% of Centres used the one-stage clotting assay for plasma FVIII activity monitoring, 7% the chromogenic test and 72% used both methods. Since the one-stage clotting assay underestimates rVIII-SingleChain by about 50%, the result must be multiplied by a factor of two to determine the patient’s FVIII activity level (as indicated in the product’s SPC) 4. The use of the conversion factor for rVIII-SingleChain was identified as a drawback by 23% of interviewed physicians, because of poor sensitivity to low values, occasional unexpected data or potential errors by clinicians or laboratory technicians not expert in haemophilia.

### Detailed records of patients treated with rVIII-SingleChain

To gather more in-depth information about clinical results and satisfaction with the current treatment, detailed case records of patients being treated with rVIII-SingleChain for at least 12 months were obtained by clinicians (cohort B, n=38) and by the patients themselves (cohort C, n=26). Demographic and clinical data of these subgroups of patients are reported in Table 2.

On the whole, patients receiving rVIII-SingleChain were young (mean age of 30–33 years) and regularly practicing physical activity (around 50% of them). Patients on a prophylaxis regimen were younger and more engaged in sport activities compared with patients treated on-demand.

**Table 2:** Demographic and clinical characteristics of patients analysed in detail by clinicians’ (cohort B) and patients’ (cohort C)

Characteristics	Clinicians’ point of view (cohort B)		Patients’ point of view (cohort C)	
Total				
Number of patients	38		26	
Age, mean	33 years		30 years	
Haemophilia severity				
Mild (%)	3 (8)		4 (15)	
Moderate (%)	9 (24)		6 (23)	
Severe (%)	26 (68)		16 (62)	
Treatment duration, mean	14.6 months		15.1 months	
By treatment regimen	On-demand	Prophylaxis	On-demand	Prophylaxis
Number of patients (%)	6 (16)	32 (84)	4 (15)	22 (85)
Age, mean	38 years	32 years	44 years	27 years
Weight, mean	71 kg	63 kg	82 kg	61 kg
Disease severity				
Mild (%)	3 (50)	0	2 (50)	2 (9)
Moderate (%)	1 (17)	8 (25)	1 (25)	5 (23)
Severe (%)	2 (33)	24 (75)	1 (25)	15 (68)
Sport practice*				
Yes (%)	2 (33)	14 (44)	2 (50)	13 (59)
No (%)	4 (67)	18 (56)	2 (50)	9 (41)

*Sports done: Swimming, gym, competitive football, non-competitive football, horse-riding, tennis, beach volleyball, weights, kickboxing, basketball, running, martial arts

### Clinical results with rVIII-SingleChain and switch analysis

**Fig. 2:**
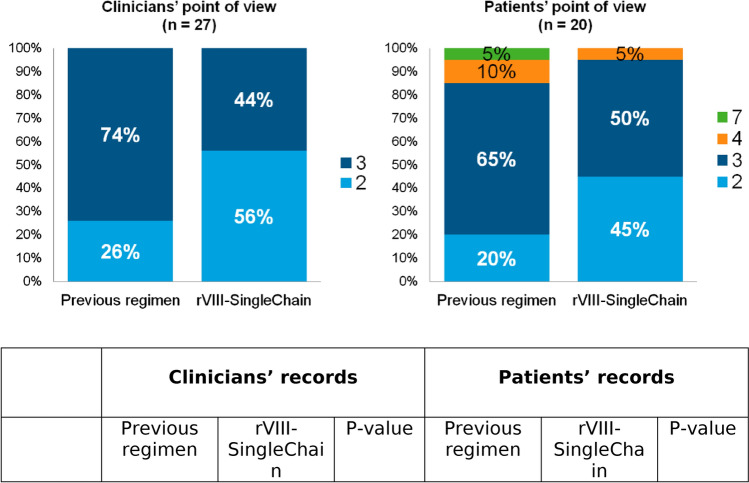
Number of weekly infusions before and after treatment with rVIII-SingleChain

Among patients reported in detail by physicians and patients (cohorts B and C), 27 and 20 patients, respectively, were currently on regular prophylaxis with rVIII-SingleChain after switching from a prophylactic regimen with another drug. A switch analysis, to assess bleeding rate and schedule of the rVIII-SingleChain treatment as compared with the previous one, was performed on these patients. Prior to the switch, the majority of them (74% of patients in the physicians’ population and 80% in the patients’ population) were on a ≥ 3 weekly dosing regimen. After patients had switched to prophylaxis with rVIII-SingleChain, these proportions were reduced to 44% and 55%, respectively (Fig. 2). As compared to the prior regimen, the proportion of patients able to be treated with a 2 weekly administration schedule increased from 26–56% in the reported population and from 20–45% in the patients’ population. Mean weekly infusions was significantly reduced from 2.74 before to 2.44 after switching to rFVIII-SingleChain in the physicians’ population (p = 0.013). A non-significant reduction, from 3.10 to 2.60, was observed in the patients’ population (p = 0.885); in this cohort, the high SD value and the small sample size may have hindered significance.

**Table:** 

	Clinicians’ records			Patients’ records		
	Previous regimen	rVIII-SingleChain	p-value	Previous regimen	rVIII-SingleChain	p-value
Mean (SD)	2.74 (0.447)	2.44 (0.506)	0.013	3.10 (1.071)	2.60 (0.598)	0.885
Median	3.0	2.0		3.0	3.0	

The reduction in weekly infusions was obtained without a parallel increase in FVIII dosage: the mean annual dosage of rVIII-SingleChain was 4320 IU/Kg (median: 4320 IU/Kg; range: 2215–6646 IU/Kg) in the 27 patients included in the clinicians’ detailed population versus 4740 IU/Kg (median: 4500 IU/Kg; range: 2215–7200 IU/Kg) with the previous drug (p = 0.178). The corresponding mean dosage per single infusion were 37 IU/Kg (median: 38 IU/Kg) versus 36 IU/Kg (median: 35 IU/Kg) (p = 0.792). After switching to rVIII-SingleChain, thus, HA patients received a lower number of FVIII administrations without significant changes in single dosages, with a resulting reduced annual consumption of FVIII.

The bleeding rate of rVIII-SingleChain on prophylaxis also significantly improved as compared with the previous treatment in the physicians’ and patients’ records, as shown in Table 3. The mean ABR in the last 12 months of treatment with rVIII-SingleChain was less than 1 in both the clinicians’ and patients’ reported populations, versus more than 2 during the last 12 months with the previous prophylaxis regimen (mean 0.96 vs. 2.15 and 0.71 vs. 2.46 events/year; p = 0.031 and p = 0.018, respectively). The percentage of patients with zero bleeds during one year of rVIII-SingleChain treatment was 52% and 40%, respectively, in the doctors’ and patients’ detailed populations, compared to 26% and 15% with the previous drug. Similar trends were observed for joint bleedings: 52% and 60% of patients reported zero joint bleeds after 12 months of rVIII-SingleChain prophylaxis in the doctor’s and patients’ populations, respectively, versus 37% and 15% with the previous treatment. The mean AJBR significantly lowered from 1.70 to 0.87 (p = 0.039) in the physicians’ cohort and from 1.90 to 0.41 (p = 0.013) in the patients’ cohort.

**Table 3:** Bleeding rates before and after treatment with rVIII-SingleChain (no. total and joint bleeds in the last 12 months of treatment with the previous drug and in the last 12 months of treatment with rVIII-SingleChain)

	Number (%)					
	Clinicians’ point of view (cohort B) (n = 27)			Patients’ point of view (cohort C) (n = 20)		
	Previous treatment	rVIII-SingleChain	p-value	Previous treatment	rVIII-SingleChain	p-value
Total bleeds/year						
Can’t recall	7 (26)	4 (15)	p = 0.031	7 (35)	3 (15)	p = 0.018
> 3	4 (15)	2 (7)		3 (15)	0 (0)	
1–3	9 (33)	7 (26)		7 (35)	9 (45)	
0	7 (26)	14 (52)		3 (15)	8 (40)	
Mean (SD)	2.15 (2.601)	0,96 (1.745)		2.46 (2.727)	0.71 (0.849)	
Range	0.0, 8.0	0.0, 6.0		0.0, 10.0	0.0, 3.0	
Median (IQR)	1.0 (3)	0.0 (1)		2.0 (2)	1.0 (1)	
Joint bleeds/year						
Can’t recall	7 (26)	4 (15)	p = 0.039	10 (50)	3 (15)	p = 0.013
> 3	4 (15)	2 (7)		2 (10)	0 (0)	
1–3	6 (22)	7 (26)		5 (25)	5 (25)	
0	10 (37)	14 (52)		3 (15)	12 (60)	
Mean	1.70 (2.430)	0.87 (1.604)		1.90 (2.079)	0.41 (0.712)	
Range	0.0, 8.0	0.0, 6.0		0.0, 6.0	0.0, 2.0	
Median (IQR)	0.5 (3)	0.0 (1)		1.5 (2)	0.0 (1)	

### Satisfaction with rVIII-SingleChain: the clinicians’ point of view

According to clinicians’ responses after one year of observation, the most convincing features of rVIII-SingleChain were the longer half-life as compared to traditional rFVIII (36%), the efficacy (29%) and the safety (21%), albeit some of them pointed out the need for longer follow-ups to drive definitive conclusions. The need for a conversion factor for measuring FVIII levels (29%) and the issues associated to laboratory monitoring and dosages (14%) were reported as the less convincing features. 36% of interviewed doctors did not find any drawbacks with the use of rVIII-SingleChain.

In general, mean satisfaction ratings were quite high among physicians: 4.14 after one year, with 86% of clinicians affirming to be satisfied or very satisfied (57% and 29%, respectively) of their experience with rVIII-SingleChain, whereas 14% were neither satisfied nor unsatisfied. The main reasons for clinicians’ satisfaction about their patients in prophylaxis with rVIII-SingleChain were: excellent responses/efficacy (25%), bleeding reduction/no bleeding (22%), greater compliance (19%), safety (16%), and fewer infusions (13%).

### Satisfaction with rVIII-SingleChain: the patients’ point of view

Overall, patients’ satisfaction was comparable to that reported by clinicians: 86% of them declared to be satisfied/very satisfied (54% and 32%, respectively) and 14% to be neither satisfied nor unsatisfied. Mean satisfaction rating was 4.18. Notably, none of them affirmed to be not at all satisfied. Main satisfaction reasons reported by the patients were linked to both emotional wellbeing aspects (“I feel more protected”, 41%; “I’m calm/I feel better”, 32%) and to the drug’s efficacy features (“I have reduced my number of infusions”, 32%; “positive continuity with the previous therapy”, 18%). 68% of patients did not find any area of dissatisfaction, whereas 32% of them mentioned areas of lower satisfaction mainly linked to the general burden of the disease (need for intravenous infusions, everyday life problems, and difficulties with the injections).

**Fig. 3:**
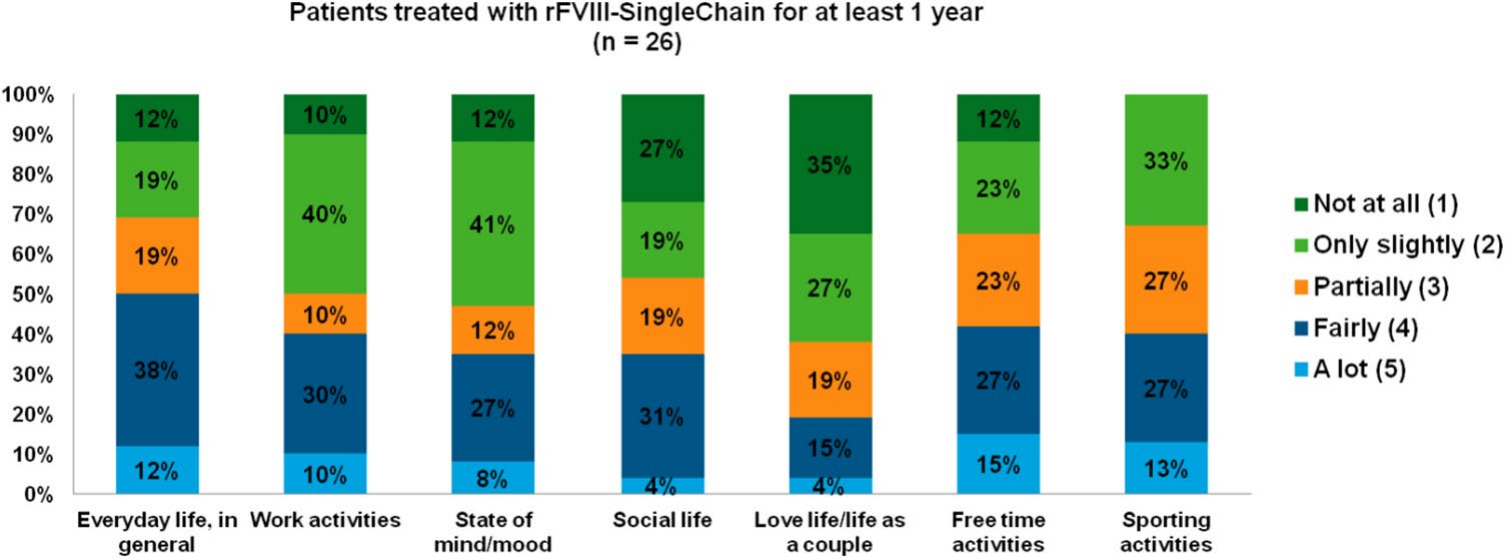
Impact of haemophilia on everyday life from the patients’ perspective

The last part of patients’ interview was dedicated to explore their general experience with haemophilia and the level of information on the disease. Required to describe their feelings about haemophilia, 35% of patients judged their daily life normal, and 23% to have to pay more attention, while 23% felt limited in life and 23% reported feelings of fear, anxiety, worry, and depression. Although prophylactic therapy was judged to confer more protection in everyday life and sport (45% of patients) and higher freedom (30%), 50% of patients still complained about the dependency on continuous infusions. Patients were requested to indicate how much illness interfered with their everyday life and with specific aspects of their life: the results are illustrated in Fig. 3. Among 26 patients treated with rVIII-SingleChain for at least 1 year (cohort C), free time and sporting activities were the aspects of life most affected by the disease, whereas globally half of them (50%) reported no or partial impact on everyday life, in general.

Overall, all the patients declared to have a good level of information on their disease and its treatments (85% judged themselves fairly informed, and 15% very informed), mainly acquired by their treating physician (92%). Other sources of information were websites/internet forums (58%) and patients’ associations (54%). A good degree of satisfaction was reported by patients with the services offered by their HTC (mainly availability of medical staff in case of emergency, relationship with the doctor, and time dedicated to the patients), but expectations for improvements in services were still present: the most interesting services, not offered by the Centre, were the home delivery of drugs (46%) and an office to handle bureaucratic matters (27%).

## Discussion

Our survey explored physicians’ and patients’ points of view and clinical experience after switching to rVIII-SingleChain for the treatment of HA. The results reinforced the good clinical performances of rVIII-SingleChain as previously reported in the pivotal clinical trials, namely the significant proportion of patients able to follow a twice per week prophylaxis regimen, without an increase in annual total FVIII consumption, and reduced ABRs and AJBRs. Moreover, the satisfaction ratings with rVIII-SingleChain were particularly elevated, highlighting treatment’s efficacy and safety among the doctors and the positive impact on everyday life and emotional wellbeing among the patients.

The population described in our analysis included prevalently young patients with HA (mean age of about 30 years) actively engaged in physical activity, 73% of whom had a severe form of the disease and were treated on prophylaxis (94% of severe patients). Prophylaxis is recommended in patients with severe HA by current guidelines [10, 11], but limited data are available in Europe about its actual prescription in the clinical management of haemophilia patients [12]. The data obtained by our national sample suggest that prophylaxis is in fact the preferred treatment regimen in young severe HA patients in the Italian everyday clinical practice. 98% population enrolled in this survey had received previous treatment before switching to rVIII-single chain therapy. Among the enrolled patients, about 60% underwent previous treatment, no longer available on the market, therefore clinicians opted for rVIIISingle Chain for its efficacy and safety features.

Compared to the previous treatment, a significant reduction in the number of weekly infusions was reported in patients receiving prophylaxis with rVIII-SingleChain: 56% of patients were able to follow a twice a week injection regimen as compared to 26% with the previous drug (clinicians’ detailed population). Burden of care is a highly relevant factor associated with prophylaxis treatment in haemophilia patients; our data suggest that in a clinical practice setting, more than half of HA patients treated with rFVIII-SingleChain, including the majority of patients with severe disease, can be safely and effectively dosed with a twice a week prophylactic regimen, with an overall reduction of treatment burden as compared to other full length rFVIII products.

In the face of a reduced number of injections, no increases in FVIII consumption was observed; in fact a non-significant reduction in the patient’s mean annual dosage was reported in the switch analysis of prophylaxis patients. Notwithstanding the lack of statistical significance, likely due to the small sample size as a major factor, the net numerical difference (29.400 IU/year for a patient with a body weight of 70 kg) may have very relevant economic implications for national health care systems when costs associated with haemophilia care are considered. These data are especially important since a higher consumption and expenditure have been reported in patients who had switched from an SHL to an EHL product in a real-world analysis (not including rVIII-SingleChain) [13], and confirm the improved PK parameters showed by rVIII-SingleChain as compared to classical SHL products [5]. It must be noted that the majority (66%) of the 35 patients currently on prophylaxis with rVIII-SingleChain and previously treated with prophylaxis used 2nd generation recombinant FVIII and none of them used EHL products. Although rVIII-SingleChain was not strictly classified as a long-acting rFVIII product [14], improved PK parameters have been demonstrated versus standard half-life products (i.e. octocog alfa) [5, 15]. Our findings confirm the clinical relevance of these observations and suggest a longer half-life of rVIII-SingleChain compared to classical SHL products in the clinical setting.

Low ABRs were reported by clinicians and patients treated with rVIII-SingleChain in our survey, with a mean of 0.96–0.71 (respectively) events observed in 1 year of treatment. By comparison, the mean ABRs reported by doctors and patients with the previous drug were 2.15 and 2.46, suggesting an improved bleeding control associated to rVIII-SingleChain prophylaxis. Similar data were observed in relation to joint bleedings, whose annual rates showed a decrease as compared with the previous treatment in the physicians’ and patients’ reports. Overall, 52–60% of prophylaxis patients (clinicians’ and patients’ cohorts, respectively) did not reported any joint bleeds after one year of rVIII-SingleChain treatment, as compared to 37% and 15%, respectively, with the previous products. Prevention of arthropathy and improvement of QoL are the main goals of prophylaxis in severe HA patients: in the pivotal rVIII-SingleChain trials median overall ABRs of 1.14 and 3.69 were observed in severe adult and children patients, respectively [7, 8]. The low bleeding rates reported in our clinical practice survey demonstrate that an efficient control of bleeding events can be obtained with rVIII-SingleChain in association with a simplified prophylactic regimen and reduced infusion frequency in a real-world setting.

Reduction in weekly infusions and high haemostatic efficacy in patients receiving prophylaxis with rVIII-SingleChain have recently been confirmed also in two European and American real-world studies. Olivieri et al. demonstrated that in a population of 225 male HA German patients, after switching to rFVIII-SingleChain prophylaxis, the mean FVIII consumption was reduced by 32% compared with prior FVIII products and the percentage of patients receiving ≤ 2 infusions/week increased from 0 to 71.4%. Excellent bleeding protection was reported, with similar or potentially lower bleeding rates than standard-acting FVIII therapies and a proportion of patients with zero spontaneous bleeds after switching to prophylaxis with rFVIII-SingleChain improved from 76.2–95.2% [9]. Similar data were reported in a real-world analysis of 120 male US patients receiving prophylaxis with three different EHL FVIII products, including rFVIII-SingleChain, rFVIIIFc, or PEG-rFVIII: prophylaxis with rFVIII-SingleChain presented lower mean factor consumption than both the other two FVIII products (− 11% and − 13.7%, respectively), with comparable bleed rates [6]. Our data support these findings.

Real-world studies are useful to validate clinical trials’ results in the daily clinical practice, a setting displaying different issues and problems. Moreover, they allow insights on personal experience, level of satisfaction and critical areas amid health workers and patients under everyday conditions. The double perspective, the medical point of view of the clinicians and the experiential perspective of patients who have to daily manage this disease, was a distinct feature of our work. Prophylactic treatment of haemophilia, although effective, imposes a significant burden of care on patients’ everyday life. Patients’ distress and the presence of anxiety and depression are key factors in the relationship of haemophilia patients with their disease and may influence adherence to replacement therapy [16, 17], shaping a point of view on the illness potentially different from that of the specialists involved in their care. Notably, although the two detailed populations analysed in our study were not completely superimposable, and thus not directly comparable, patients’ reports appeared substantially in accord with the vision of their physicians. Both doctors and patients, when requested to compare rVIII-SingleChain prophylaxis with the previous treatment, identified the number of bleeds and the reduction in injection frequency as areas of improvement. Satisfaction rates were also comparable in the two populations, reaching more of 85% of satisfied/very satisfied among both clinicians and patients. However, some distinction between doctors’ and patients’ points of view became evident in the analysis of the main reasons of satisfaction. Whereas clinicians mainly underscored efficacy/safety features of rVIII-SingleChain, patients prevalently highlighted the positive effect on their QoL and emotional wellbeing. Patients reported a reduction in the impact of haemophilia on their work life, their state of mind and mood, and on their general everyday life. Positive expression such as “protection” and “peace-of-mind” could be found among patients’ spontaneous comments regarding their experiences with haemophilia. The relevance of these findings may be significant in the clinical practice, since “patients’ acceptance” and “compliance to therapy” were described within the major barriers to prophylaxis in haemophilia patients. On the contrary, “bleeding frequency” and “presence of target joints” were factors favouring the prescription of prophylaxis among clinicians [12]. Overall, doctors’ and patients’ perceptions may remain different, because different are the main focuses and expectations (an effective and safe treatment versus a good QoL and perception of being protected against the disease). These considerations underline the value of the matching assessment and satisfaction on rVIII-SingleChain reported by clinicians and patients in the present survey, suggesting that the drug is able to favourably conjugate different points of view on haemophilia prophylactic treatment. Reducing the burden associated with prophylaxis while maintaining a high haemostatic protection without FVIII increased consumption might significantly improve clinical outcomes and QoL in haemophilia patients.

The main limitations of the current survey are related to the small sample size and to the limits of the tool itself. Although representative of the entire national condition, our specialist sample may suffer of potential selection bias. At the same time, since the participation of patients was voluntary, we were not able to completely match the clinicians’ and patients’ reported populations. Although a limit, this issue might also allow for an independent comparison between two different points of view, which, as already reported, appeared to be strikingly similar and complementary in their dissimilarities. In the interpretation of the results, surveys, as every self-reported outcome, may suffer of a lack of objectivity. On the other side, they offer a unique opportunity to collect the point of view of all the actors involved. We are well aware that our findings cannot substitute an observational study, but also believe that self-reported data have a value specifically related to their subjectivity. While acknowledging the limitation of this study, the results presented here show that both clinicians and patients felt that switching to rVIII-SingleChain reduced treatment burden and bleeding events, increasing their satisfaction with prophylactic haemophilia treatment, a finding worth to be kept in mind in the choice of therapeutic options for this disease.

In conclusion, this study evaluated the benefits associated with rVIII-SingleChain treatment in a cohort of specialists and patients from HTCs in Italy.

Results of this real-world study for rVIII-SingleChain support data reported in the clinical studies [18]. The survey results highlight the clinicians’ indication of rVIIISingle Chain as a reliable choice with a favorable pharmacokinetic profile. Responses to the survey were based on the treatment efficacy (evaluated as bleeding reduction or absence of bleeding), safety, and favorable half-life.

The analysis of their clinical experience suggests that switching to rVIII-SingleChain allowed patients to reduce their dosing frequency without increasing factor consumption or compromising clinical outcome. Both clinicians and patients reported high levels of satisfaction with the treatment, pointing out its efficacy and positive impact on everyday life and emotional wellbeing.

GF, AF, PG, ML, GM, MM, ACM, BP, MS and EZ declare that they have no commercial association(e.g. consultancies, stock ownership, equity interest, patent/licensing arrangements etc.) that might pose a conflict of interest in connection with the submitted article.
